# Tendon mechanical properties are enhanced *via* recombinant lysyl oxidase treatment

**DOI:** 10.3389/fbioe.2022.945639

**Published:** 2022-08-05

**Authors:** Phong K. Nguyen, Aniket Jana, Chi Huang, Alison Grafton, Iverson Holt, Michael Giacomelli, Catherine K. Kuo

**Affiliations:** ^1^ Department of Biomedical Engineering, University of Rochester, Rochester, NY, United States; ^2^ Center for Musculoskeletal Research, University of Rochester Medical Center, Rochester, NY, United States; ^3^ Fischell Department of Bioengineering, University of Maryland, College Park, MD, United States; ^4^ Department of Orthopaedics, University of Rochester Medical Center, Rochester, NY, United States; ^5^ Department of Orthopaedics, University of Maryland School of Medicine, Baltimore, MD, United States

**Keywords:** tendon, collagen crosslinking, lysyl oxidase, tendon healing, mechanical properties, birth deformity, orthopaedic, musculoskeletal

## Abstract

Tendon mechanical properties are significantly compromised in adult tendon injuries, tendon-related birth defects, and connective tissue disorders. Unfortunately, there currently is no effective treatment to restore native tendon mechanical properties after postnatal tendon injury or abnormal fetal development. Approaches to promote crosslinking of extracellular matrix components in tendon have been proposed to enhance insufficient mechanical properties of fibrotic tendon after healing. However, these crosslinking agents, which are not naturally present in the body, are associated with toxicity and significant reductions in metabolic activity at concentrations that enhance tendon mechanical properties. In contrast, we propose that an effective method to restore tendon mechanical properties would be to promote lysyl oxidase (LOX)-mediated collagen crosslinking in tendon during adult tissue healing or fetal tissue development. LOX is naturally occurring in the body, and we previously demonstrated LOX-mediated collagen crosslinking to be a critical regulator of tendon mechanical properties during new tissue formation. In this study, we examined the effects of recombinant LOX treatment on tendon at different stages of development. We found that recombinant LOX treatment significantly enhanced tensile and nanoscale tendon mechanical properties without affecting cell viability or collagen content, density, and maturity. Interestingly, both tendon elastic modulus and LOX-mediated collagen crosslink density plateaued at higher recombinant LOX concentrations, which may have been due to limited availability of adjacent lysine residues that are near enough to be crosslinked together. The plateau in crosslink density at higher concentrations of recombinant LOX treatments may have implications for preventing over-stiffening of tendon, though this requires further investigation. These findings demonstrate the exciting potential for a LOX-based therapeutic to enhance tendon mechanical properties *via* a naturally occurring crosslinking mechanism, which could have tremendous implications for an estimated 32 million acute and chronic tendon and ligament injuries each year in the U.S.

## Introduction

Tendon is a highly collagenous connective tissue that transmits forces from muscle to bone to enable movement. Unfortunately, tendon injuries are highly prevalent throughout the body. For example, flexor tendon injuries affect 33 per 100,000 persons each year (de Jong et al., 2014). Achilles tendon injuries account for up to 50% of all sports-related injuries (Järvinen et al., 2005; Raikin et al., 2013). Rotator cuff injuries affect 30% of people over the age of 60, leading to over 600,000 repairs each year in the U.S. (Vitale et al., 2007; Dang and Davies, 2018). Despite their critical musculoskeletal functions, tendons lack the ability to regenerate when injured (Arya and Kulig, 2010; Helland et al., 2013; Derwin and Thomopoulos, 2020; Sarmiento and Little, 2021). Tendons are also commonly associated with heritable connective tissue disorders and birth abnormalities, including Ehlers-Danlos syndrome (EDS) and clubfoot (Browne, 1931; Windisch et al., 2007; Gazit et al., 2016). It has been estimated that tendon-related birth defects affect up to 1 in 100 live births each year (Kowalczyk and Feluś, 2016). Unfortunately, there are currently no effective treatment strategies to restore native mechanical properties to tendons in any of these scenarios. Thus, there is a critical need for therapeutics to promote tendon mechanical properties.

Lysyl oxidase (LOX) is a critical regulator of tendon mechanical properties during new tissue formation. LOX catalyzes the formation of crosslinks between collagen molecules, including trivalent hydroxylysyl pyridinoline (HP) and lysyl pyridinoline (LP), the two most prevalent LOX-mediated collagen crosslinks in tendon (Eyre et al., 1984; Eyre et al., 2008). We previously discovered that inhibition of LOX activity during tendon development blocks formation of new HP and LP crosslinks between collagen molecules, and that this inhibition of collagen crosslinking prevents increases in tendon elastic modulus (Marturano et al., 2013; Marturano et al., 2014). Furthermore, inhibition of increases in elastic modulus due to inhibition of LOX crosslinking occurred without affecting collagen content and organization (Marturano et al., 2013; Marturano et al., 2014). Our studies also revealed that tendon elastic modulus correlates strongly with crosslink density (*r* ^2^ = 0.8, *p* < 0.0001) as well as LOX activity levels (*r* ^2^ = 0.8, *p* < 0.05) during normal development and also when LOX activity is inhibited (Marturano et al., 2014; Pan et al., 2018). Taken together, LOX plays an important role in regulating tendon mechanical properties by modulating collagen crosslinking.

LOX-mediated collagen crosslink density correlates significantly with elastic modulus in healthy adult tendons in human, goat, and horse (Eyre et al., 1984; Ng et al., 1996; Thorpe et al., 2010). Unfortunately, adult tendons post-healing are associated with abnormal and typically inferior mechanical properties (Beredjiklian et al., 2003; Arya and Kulig, 2010; Helland et al., 2013; Sereysky et al., 2013; Theodossiou and Schiele, 2019; Arvind and Huang, 2021). Higher HP and LP crosslink density levels have been reported during the early stages of healing of injured human supraspinatus tendons compared to healthy tendons, but the study only examined one timepoint and it was hypothesized that these increases were temporary (Bank et al., 1999). Indeed, rabbit medial collateral ligaments have been reported to possess lower elastic modulus and pyridinoline crosslink density after complete healing at 40 weeks post-injury compared to non-injured controls (Frank et al., 1995). In addition, it has been shown in skin and liver that crosslink density increases significantly during the early remodeling phase but eventually decreases and remains at lower levels compared to healthy controls (Bailey et al., 1975; Bailey and Light, 1985; Ricard-Blum et al., 1992). Taken together, enhancement of LOX-mediated collagen crosslinking may be necessary to restore normal tendon mechanical properties and function.

Crosslinking has been studied as a potential treatment to enhance tendon mechanical properties with limited degrees of success (Caliari et al., 2011; Fessel and Snedeker, 2011; Fessel et al., 2012; Fessel et al., 2014; Green et al., 2017). In particular, crosslinkers such as 1-ethyl-3-(3-dimethylaminopropyl) carbodiimide, glutaraldehyde, and genipin have been tested to enhance the mechanical properties of tendon explants and engineered tendon tissue (Caliari et al., 2011; Fessel and Snedeker, 2011; Fessel et al., 2012; Green et al., 2017). However, these crosslinkers do not occur naturally in human and are associated with cytotoxicity at levels that improve tendon mechanical properties (Fessel et al., 2014). In contrast, LOX-mediated crosslinking of collagen occurs naturally during development and correlates directly with mechanical properties of homeostatic adult tendons (Eyre et al., 1984; Ng et al., 1996; Thorpe et al., 2010). A therapeutic treatment that promotes naturally occurring crosslinking to enhance tendon mechanical properties would have significant clinical implications.

In addition to poor tendon healing, abnormal LOX-mediated collagen crosslinking has been implicated in heritable connective tissue disorders and birth abnormalities that affect tendon, including EDS, clubfoot, and Menkes disease (Di Ferrante et al., 1975; Kuivaniemi et al., 1985; Royce and Steinmann, 1990; Knitlova et al., 2021; Novotny et al., 2022). For example, EDS is associated with lower LOX enzyme levels and compromised tendon mechanical properties compared to healthy counterparts (Kuivaniemi et al., 1985; Nielsen et al., 2014). In clubfoot patients, LOX protein and LOX-mediated crosslink density levels are both higher in the contracted tendons as compared to the non-contracted tendons (Knitlova et al., 2021; Novotny et al., 2022). Although clubfoot is treatable postnatally *via* an extensive 5-year bracing process called the Ponseti method, the treated clubfoot tendons possess lower elastic modulus compared to age-match healthy children and recurrence of clubfoot after the 5-year process is as high as 37% (Richards et al., 2008; Bor et al., 2009; Masala et al., 2012; Sætersdal et al., 2012; Zhao et al., 2014). These examples indicate a need for more effective treatments that can restore native mechanical properties of tendon that are involved in connective tissue disorders and musculoskeletal birth defects. Based on these findings, future therapeutics could target LOX-mediated collagen crosslinking to enhance mechanical properties of abnormally weak tendons during treatment of connective tissue disorders and musculoskeletal abnormalities in childhood years.

There is a critical need to effectively enhance tendon mechanical properties of abnormally weak tendons that are associated with connective tissue disorders and birth abnormalities and adult tendon injuries. Because tendon-related birth defects develop at different stages during pregnancy and injured adult tendons undergo various stages of new tissue formation during healing, we aimed to examine the effects of recombinant LOX (rLOX) treatment on embryonic tendons at different developmental stages. We treated chick embryo tendons from two developmental stages with rLOX and measured LOX-mediated collagen crosslink density, tendon mechanical properties, cell number and viability, and collagen content, organization, and maturation. We hypothesized that 1) rLOX treatment would enhance LOX-mediated collagen crosslink density and tendon mechanical properties at different developmental stages, 2) LOX-mediated collagen crosslink density and tendon elastic modulus would plateau at higher rLOX concentrations, and 3) cell viability and collagen content, organization, and maturation would not be affected by rLOX treatment. Our findings here establish the feasibility of therapeutic treatment strategies to enhance tendon mechanical properties by promoting LOX-mediated collagen crosslinking, a naturally occurring mechanism.

## Materials and methods

### Experimental overview

Chick embryo calcaneal tendons from two developmental stages Hamburger-Hamilton stage (HH) HH40 (embryonic day 14) and HH43 (embryonic day 17), were used for *in ovo* and *ex ovo* experiments. HH40 and HH43 were chosen based on our previous findings that significant collagen deposition, organization, and crosslinking, as well as elaboration of tendon mechanical properties, are rapidly increasing through HH40 and HH43 (the embryo hatches after HH45) (Kuo et al., 2008; Marturano et al., 2013; Marturano et al., 2014). For *in ovo* experiments, recombinant (r) LOX or vehicle (Ctrl) was injected *in vivo* between the calcaneal tendon and the underlying tibia bone. Embryos were sacrificed and tendons were dissected for tensile testing after 48 h. For *ex ovo* experiments, tendon explants were cultured in medium supplemented with rLOX or vehicle. Tendon explants were harvested after 72 h culture for atomic force microscopy, histological staining, and two-photon imaging. The number of biological replicates in all experiments was determined by power analysis, where each sample was from a different chick embryo.

### *In ovo* culture

Fertilized white leghorn chick embryos (University of Connecticut Poultry Farm, CT) were cultured in humidified rocking incubators at 37.5°C. At HH20, a small window was created in the eggshell, as we previously described (Stein et al., 2019). Transparent tape was placed on top of the window and the windowed eggs were subsequently incubated in humidified non-rocking incubators at 37.5°C. At HH38 and HH41, embryos were each injected with 3 µg of rLOX (Origene, MD) dissolved in 30 µl vehicle (25 mM Tris HCl, pH 7.3, 100 mM glycine, 10% glycerol) between the calcaneal tendon and the underlying tibia bone at 0 h and 24 h. The contralateral control limb was injected with 30 µl vehicle. The window was re-sealed with tape and embryos were returned to the incubators. After 48 h, embryos were sacrificed at HH40 and HH43, and the calcaneal tendons were dissected for tensile mechanical testing.

### Tensile mechanical testing and data analysis

Tendons treated *in vivo* with either vehicle or rLOX were harvested and tensile tested as previously described (Navarro et al., 2022). Briefly, tendons were gripped at each end and stretched uniaxially at 1% strain per second until failure. Elastic modulus was calculated based on the slope of the linear region of the stress-strain curve. Peak stress was calculated by normalizing the maximum load recorded during testing to the cross-sectional area. Peak strain was determined by the strain value at peak stress.

### *Ex ovo* explant culture

Calcaneal tendons of HH40 and HH43 chick embryos were dissected out of the limb, using our previously established protocol to ensure muscle and fibrocartilage were excluded (Navarro et al., 2022). After the tendon was separated from the limb, tendon bundles separated easily from each other during gentle extraction using fine tweezers. Tendon bundles range from 1.55 to 3.41 mm in length and from 200 to 400 µm in diameter. Each experiment required *N* = 5 biological replicates, with a different embryo as the source of each biological replicate (N). Bundles from a tendon of the same embryo were divided across treatment groups: Ctrl, 1X, 2X, and 5X rLOX. This process was implemented for *N* = 5 different embryos. Tendon bundle explants were cultured for 72 h in complete medium consisting of Dulbecco’s modified Eagle’s medium (Life Technologies, CA), 10% fetal bovine serum (Atlanta Biologicals, GA), and 1% antimycotic-antibiotic (Gibco, CA). Culture medium was replenished and supplemented with either 0 (vehicle), 1.5, 3, or 7.5 µg/ml rLOX (corresponding to 1X, 2X, or 5X rLOX) at 0 h, 24 h, and 48 h. Studies have shown LOX remains active up to 120 h *in vitro* (Trackman et al., 1981; Kagan and Li, 2003). Tendon explants were harvested for assays at 72 h.

### Cryopreservation and cryosectioning

Tendons were cryopreserved and cryosectioned as previously described (Marturano et al., 2013). Briefly, tendons were immersed in 10% (v/v) dimethyl sulfoxide (Sigma, MO) HBSS solution for 15 min at room temperature, with tissue-to-solution volume ratio of at least 1:10. Tendons were then incubated in optimal cutting temperature (OCT) medium (Sakura Finetek, CA) for 30 min at room temperature, slowly frozen at −1°C/min to −80°C in an isopropanol bath, and then stored in a −80°C freezer. Frozen blocks were cryosectioned at 20 µm thickness for assays described below. At the start of as well as during sectioning, the frozen tissue block was adjusted as needed to ensure collagen fibers were oriented in the longitudinal direction, parallel to the sectioning plane.

### Force volume-atomic force microscopy

AFM was performed on 20 μm-thick sections following our previously established methods (Marturano et al., 2013). Briefly, tendon cryosections were immersed in PBS at room temperature for 5 min to remove the OCT and DMSO. Samples were then immersed in fresh PBS during AFM testing. To ensure the location of AFM testing was consistent from one sample to the next, we visualized the entire bundle length using brightfield imaging. The mid-point of tendon bundles along the longitudinal length was determined using the x-y coordinates provided by the imaging software and chosen as the location for AFM probing. Pyramidal Si_3_N_4_ probes with an approximately 20 nm tip radius and a spring constant of 0.06 N/m (Bruker, MA) were used on an MFP-3D AFM machine (Asylum Research, CA). Tissue was indented by the nanoscale probe at 6 µm/s rate. For each measurement, the probe was descended onto the tissue until 40 nm deflection in the cantilever was detected. Force maps were recorded within a 10 µm × 10 µm area with a 64 × 64 force curve array. Individual elastic moduli were calculated by fitting individual force-displacement curves to the Hertzian equation, also as described in our previous study (Marturano et al., 2013). For each tendon sample, median elastic modulus was calculated from 4,096 data points per force map. Median, rather than mean, was reported for each sample because of non-symmetrical increases in moduli across the 4,096 data points per force map.

### Hematoxylin and eosin staining and image analysis

Tendon sections were stained with H&E and imaged under brightfield microscopy (20X objective, ZEISS Axio Scan, Germany). For each biological replicate (N), cells were counted per 120 × 120 µm^2^ area manually using the Cell Counter plug-in in ImageJ (NIH, MD) by three independent reviewers. The cell number per area was calculated as total cells divided by area.

### TdT-mediated dUTP nick end labeling (TUNEL) staining and image analysis

Apoptotic cells were detected in TUNEL-stained tendon sections using the *In Situ* Cell Death Detection Kit, TMR red (Roche, Germany). Counter-staining with Hoechst 33342 (Thermo Fisher, MA) was used to visualize all cell nuclei. Stained sections were imaged using a FLUOVIEW FV3000 laser scanning confocal system (Olympus, PA). TUNEL-positive cells and Hoescht 33342-positive cells fluoresced red and blue, respectively. To enhance visualization in figures, Hoescht 33342-positive cells were pseudo-colored green. To measure cell viability based on TUNEL staining, images were converted to 16-bit (grey scale) and a universal threshold was applied to all images to identify individual cells. Total cell number based on Hoeschst staining (N_total_) and TUNEL-positive cells (N_a_) were counted using the Analyze Particle function in ImageJ (NIH, MD). The percentage of viable cells was calculated as [(N_total_–N_a_)/N_total_ ] x 100%.

### Picrosirius red staining and image analysis

Tendon sections were stained with PSR and imaged using brightfield and polarized light microscopy (20X objective, ZEISS Axio Scan, Germany). Light source angle was kept consistent between all samples as it is non-adjustable on the ZEISS Axio Scan. Collagen content was assessed by analyzing brightfield images of PSR-stained sections. Brightfield images were converted into 8-bit format and thresholded to identify PSR-positive staining areas. PSR-positive staining area fraction of each image was quantified to determine collagen content per unit area. Collagen maturity was assessed by analyzing polarized images of the same PSR-stained sections used in the analysis above. It has previously been shown that polarization color shifts from the shorter wavelength color green to higher wavelength colors yellow and then red as collagen fibers increase in diameter and packing density, as typically occurs during maturation (Junqueira et al., 1982; Dayan et al., 1989). Accordingly, the percentages of green, yellow, and red collagen fibers have been used to evaluate collagen maturity in polarized microscopy images of tissues (Wolman and Kasten, 1986; Monaghan et al., 2017; Patel et al., 2018). Here, we characterized green fibers as immature fibers, yellow fibers as intermediate fibers, and red fibers as mature fibers, using the following method. Polarized images were converted into 8-bit format. In order to set the appropriate threshold levels that classify red, yellow, and green pixels, we evaluated the intensity ratio of red to green signal (I_R_/I_G_) for at least 20 visibly red and green regions within tendon per embryonic stage. The selection of lower to higher intensity red and green regions was performed by 3 independent reviewers. A pixel was classified as red if I_R_/I_G_ ≥ 1.8, green if I_R_/I_G_ ≤ 1.1, and yellow if 1.1< I_R_/I_G_<1.8. The ranges of I_R_/I_G_ ratio for each color were entered into a MATLAB code. To ensure there was no human bias, the same code was applied to every image to quantify the percentage of red (mature), yellow (intermediate), and green (immature) fibers.

### Two-photon microscopy and image analysis

Tendon sections were imaged using an Olympus FVMPE-RS microscope system with an Olympus 25X water immersion objective (Olympus, Japan). Fibrillar collagen was visualized based on forward second harmonic generation (F-SHG) signal (780/370–410 nm). Image segmentation was performed by generating a binary mask using intensity thresholding with a custom MATLAB code (Mathworks, CA). Average pixel intensity of each image was quantified to determine collagen content per unit area. The Directionality plugin in FIJI (NIH, MD) was used to quantify the angle of collagen fiber orientation (JY, 2010; Sensini et al., 2018; Bah et al., 2020). First, SHG images were imported and converted into 8-bit format. Fourier spectrum analysis was then performed to create a histogram of orientation angle of collagen fibers within the images. The histogram peak represented the predominant orientation angle, and the histogram width represented the dispersion of collagen fiber orientation angles. Lower dispersion value indicated more aligned collagen fibers.

### High performance liquid chromatography

Tendon samples were flash frozen in liquid nitrogen and ground with pestle and mortar. Samples were then transferred into 1.5 ml Eppendorf tubes for long-term storage at −80°C or immediately processed for HPLC. Frozen tendon samples were lyophilized and reduced with 10 mg/ml sodium borohydride (NaBH_4_) in 1 mM sodium hydroxide (NaOH) for 2 h at room temperature. Acetic acid was added until pH = 3 was achieved to stop the reaction. Samples were hydrolyzed with 6 M hydrochloric acid at 110°C for 20 h. Samples were lyophilized until completely dried, weighed, and then reconstituted in 50 µl of 1% heptafluorobutyric acid (HFBA) HPLC-grade water. Samples were injected into a 4.6 mm × 150 mm C18 reverse phase column (Shimadzu, Japan) and analyzed for pyridinoline content (HP and LP) based on fluorescence signal (297/395 nm) (Bank et al., 1997). Crosslink density was calculated as the ratio of HP and LP content to dry mass of each sample.

### Statistical analyses

Power analysis was performed with G*Power software based on preliminary data with power = 0.9 and significance level α = 0.05 using 1 sample, 2-sided equality, and 2-sample equivalence scenarios (Faul et al., 2007). Power analysis determined *N* = 5 per embryonic stage and condition was needed for tensile testing, AFM, H&E staining and image analysis, PSR staining and image analysis, and SHG imaging and image analysis, and *N* = 3 per embryonic stage and condition was needed for HPLC. Student *t*-test was used to analyze statistical differences between rLOX and vehicle control (Ctrl) treatments for tensile and nanoscale elastic moduli, cell number and viability, and collagen content, alignment, and maturity. 1-way ANOVA followed by Tukey’s post-hoc analysis was used to analyze statistical differences in nanoscale elastic modulus and crosslink density between Ctrl, 1X, 2X, and 5X rLOX treatments. Exponential plateau fit analysis was used to examine the plateaus in elastic modulus and crosslink density with increasing rLOX concentrations. Linear regression analysis was used to examine the correlation between elastic modulus and crosslink density. All statistical analyses were performed using Graphpad Prism v8 (CA, United States).

## Results

**FIGURE 1:**
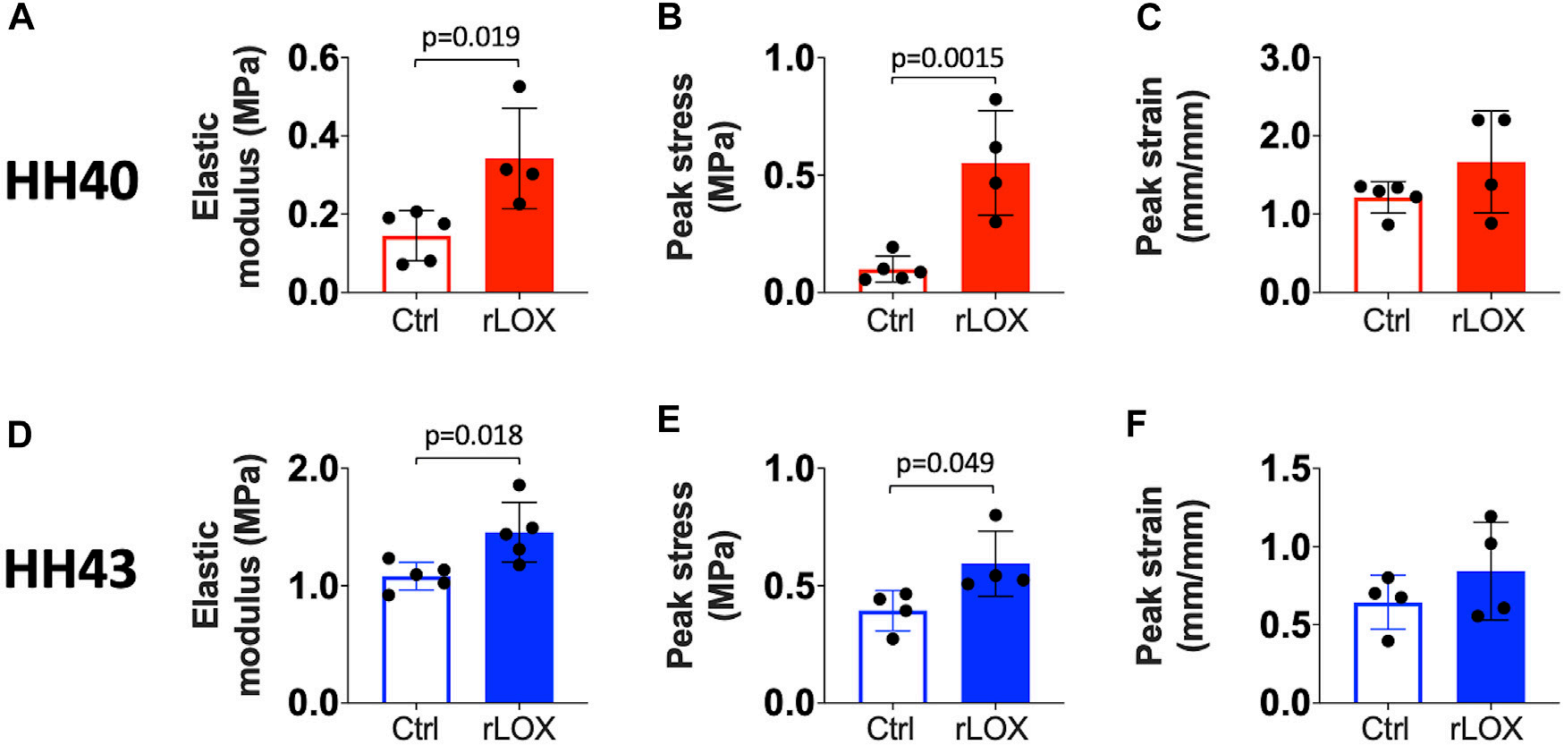
Tensile mechanical properties of HH40 and HH43 tendons were significantly enhanced by rLOX treatment of the tendon during development *in vivo*. Elastic modulus **(A)** and peak stress **(B)** of HH40 tendons increased significantly with rLOX as compared to Ctrl treatment. Peak strain **(C)** of HH40 tendons was unchanged with rLOX treatment. Elastic modulus **(D)** and peak stress **(E)** of HH43 tendons increased significantly with rLOX treatment. Peak strain **(F)** of HH43 tendons was unchanged with rLOX treatment. Statistically significant differences were determined by Student *t*-test with *p* < 0.05. *n* = 5 per stage and treatment group.

Tensile testing revealed tendon elastic modulus and peak stress increased significantly with rLOX treatment whereas peak strain was not affected for both HH40 and HH43 tendons (Figures 1A–F).

**FIGURE 2:**
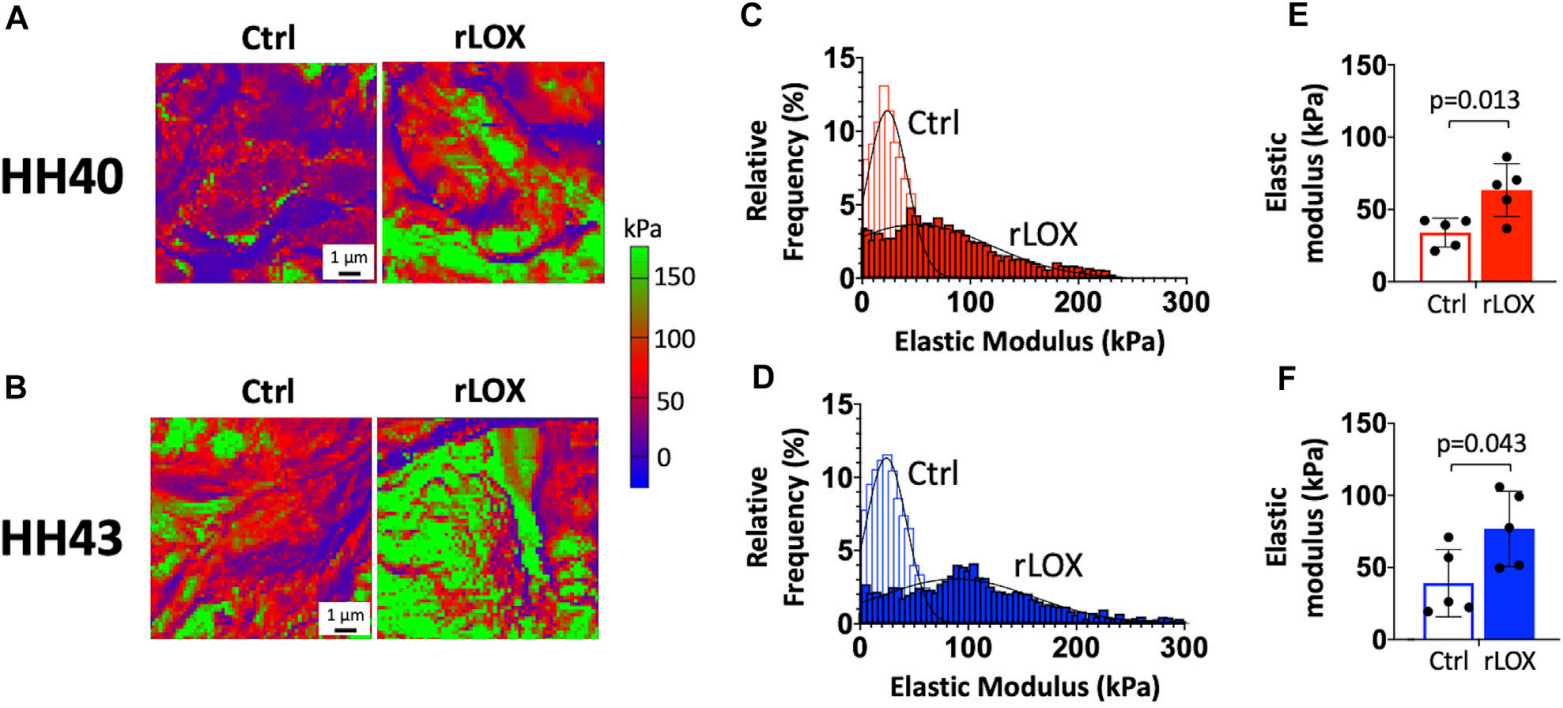
Nanoscale elastic moduli of HH40 and HH43 tendon explants were significantly increased with rLOX treatment *ex ovo*. **(A,B)** Representative elastic modulus maps showed HH40 **(A)** and HH43 tendons **(B)** possess more regions of higher elastic modulus after rLOX treatment compared to Ctrl treatment. **(C,D)** Representative histograms showed increasing frequency of higher elastic moduli in HH40 **(C)** and HH43 tendons **(D)** after rLOX treatment compared to Ctrl treatment. Additionally, treatment with rLOX led to wider distribution of elastic modulus values for tendon explants of both stages. **(E,F)** Median elastic moduli of HH40 **(E)** and HH43 tendons **(F)** were significantly increased with rLOX treatment. Statistically significant differences were determined by Student *t*-test with *p* < 0.05. *n* = 5 per stage and treatment group.

AFM maps revealed more regions with higher elastic modulus values in both HH40 and HH43 tendons when treated with rLOX (Figures 2A,B). Relative frequency histograms showed rLOX treatment led to wider distributions of elastic moduli with higher elastic modulus values (Figures 2C,D). Median nanoscale elastic moduli of HH40 and HH43 tendons both increased significantly with rLOX treatment over those with Ctrl treatment (Figures 2E,F).

**FIGURE 3:**
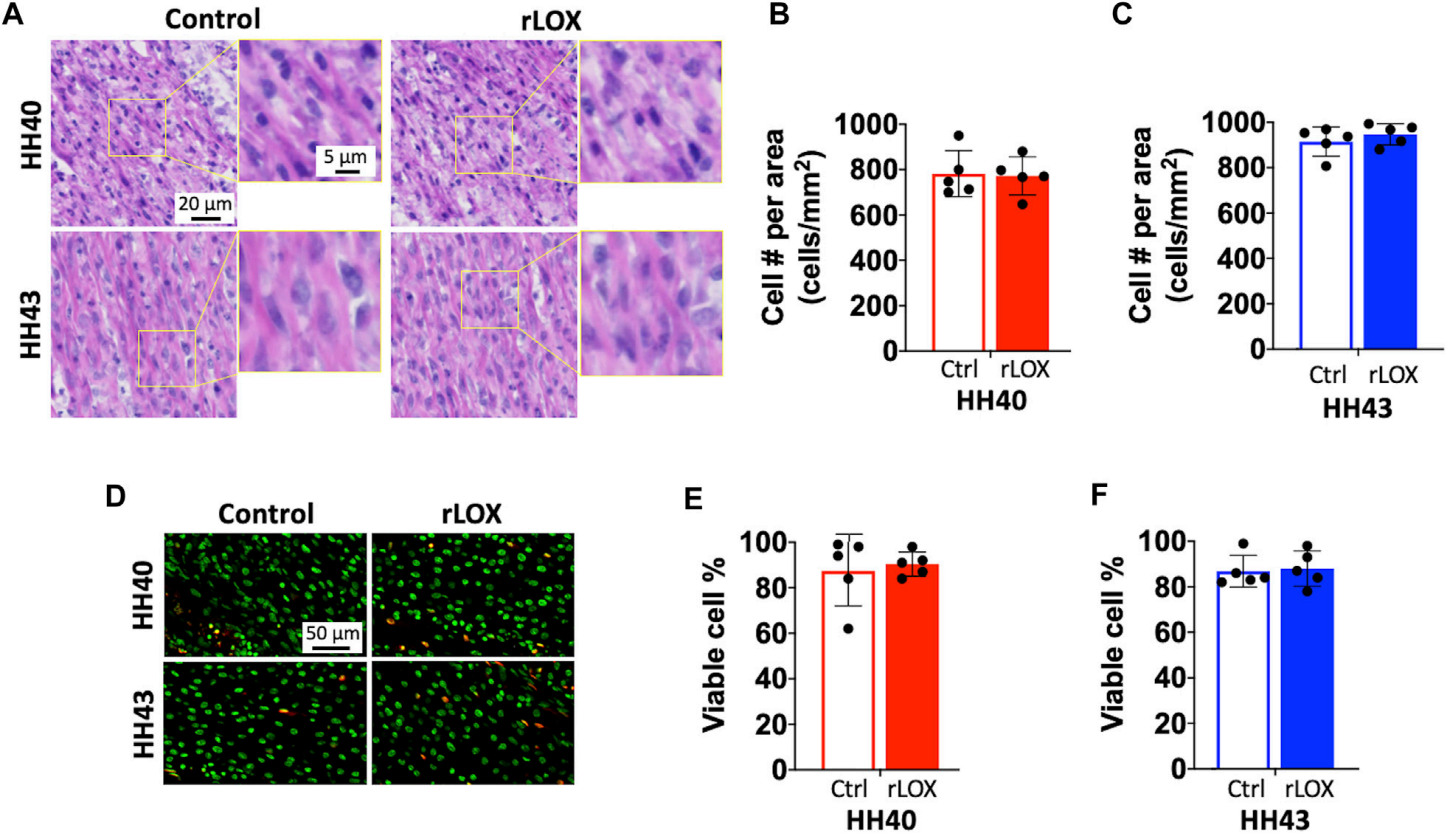
Cell number and viability of HH40 and HH43 tendon explants were similar between rLOX and Ctrl treatments. **(A)** Representative images of H&E-stained HH40 and HH43 tendons after rLOX and Ctrl treatments. High magnification images showed intact cell nuclei and lack of cell debris. **(B,C)** Cell density was not affected by rLOX treatment in both HH40 **(B)** and HH43 **(C)** tendons. **(D)** Representative images of TUNEL- and Hoescht 33342-stained HH40 and HH43 tendons after rLOX and Ctrl treatments. TUNEL-positive cells were red and Hoescht 33342-positive cells were pseudo-colored green. **(E,F)** Percentages of viable cells in both HH40 and HH43 tendons were similar between rLOX and Ctrl treatments. Statistically significant differences were determined by Student *t*-test with *p* < 0.05. *n* = 5 per stage and treatment group.

H&E staining showed intact nuclei and no cell debris in both HH40 and HH43 tendons, with no apparent differences between rLOX and Ctrl treatments (Figure 3A). Cell density remained constant between rLOX and Ctrl treatments for HH40 and HH43 tendons, quantified based on image analysis of H&E stains (Figures 3B,C). TUNEL and Hoechst 33342 staining revealed mostly viable cells with few TUNEL-positive cells in HH40 and HH43 tendons, with no apparent differences between rLOX and Ctrl treatments (Figure 3D). Percentages of apoptotic cells were similar between rLOX and Ctrl treatments, quantified based on image analysis of TUNEL-positive cells (Figures 3E,F).

**FIGURE 4:**
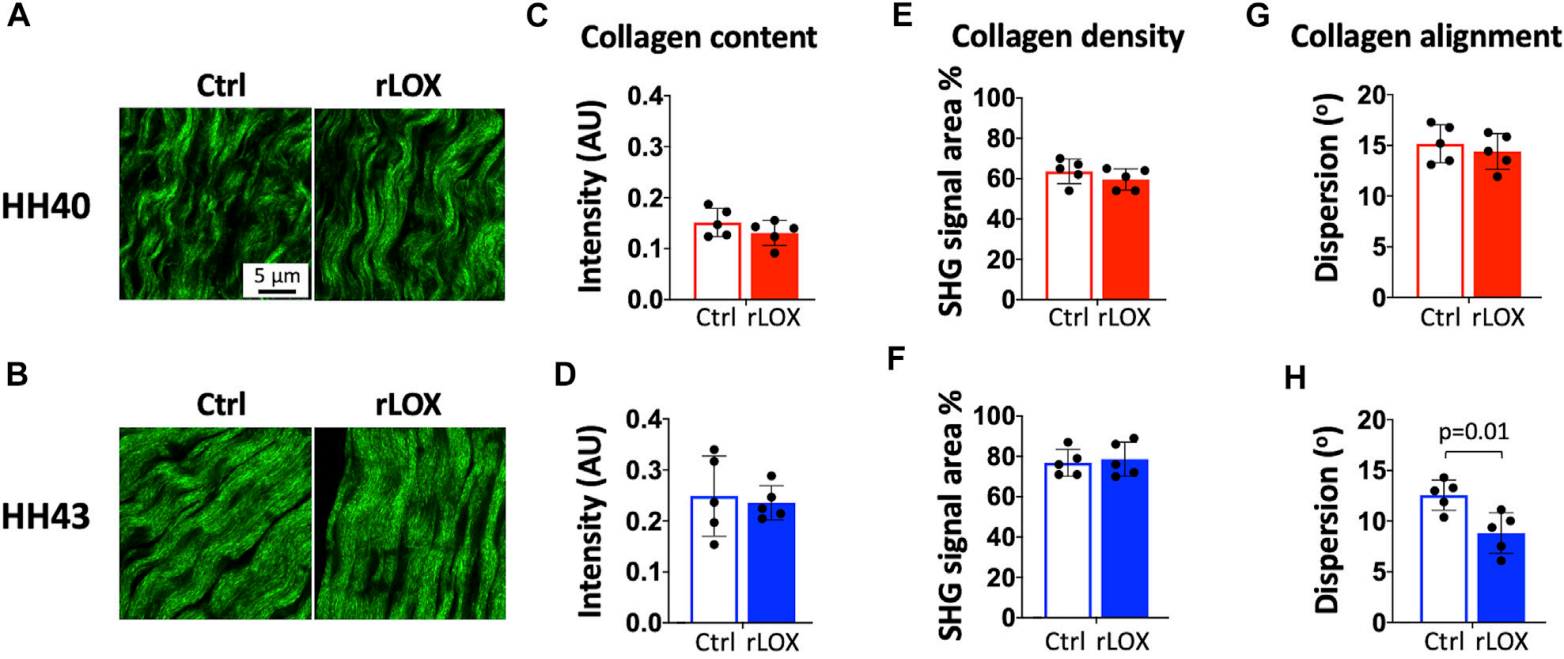
SHG imaging and image analysis of HH40 and HH43 tendon explants after rLOX and vehicle control treatments. **(A,B)** Representative SHG images of HH40 **(A)** and HH43 **(B)** tendons after rLOX and Ctrl treatments. **(C,D)** Collagen content was not affected by rLOX treatment in both HH40 **(C)** and HH43 **(D)** tendons. **(E,F)** Collagen density was not affected by rLOX treatment in both HH40 **(E)** and HH43 **(F)** tendons. **(G)** Collagen alignment, based on dispersion angle, was not affected by rLOX treatment in HH40 tendons. **(H)** Collagen alignment in HH43 tendons increased significantly with rLOX treatment, indicated by significantly lower dispersion angle in HH43 tendons after rLOX treatment. Statistically significant differences were determined by Student *t*-test with *p* < 0.05. *n* = 5 per stage and treatment group.

SHG images of fibrillar collagen appeared similar between rLOX and Ctrl treatments for HH40 and HH43 tendons (Figures 4A,B). Images were then analyzed to quantify collagen content, density, and dispersion angle. Quantification of total SHG signal intensity showed fibrillar collagen content was not affected by rLOX treatment in both HH40 and HH43 tendons (Figures 4C,D). Quantification of SHG signal density per unit area showed fibrillar collagen density was not affected by rLOX treatment in both HH40 and HH43 tendons (Figures 4E,F). Quantification of dispersion angle showed fibrillar collagen alignment was not affected by rLOX treatment in HH40 tendons (Figure 4G). However, lower dispersion angle with rLOX treatment indicated higher levels of collagen alignment with rLOX treatment in HH43 tendons (Figure 4H).

**FIGURE 5:**
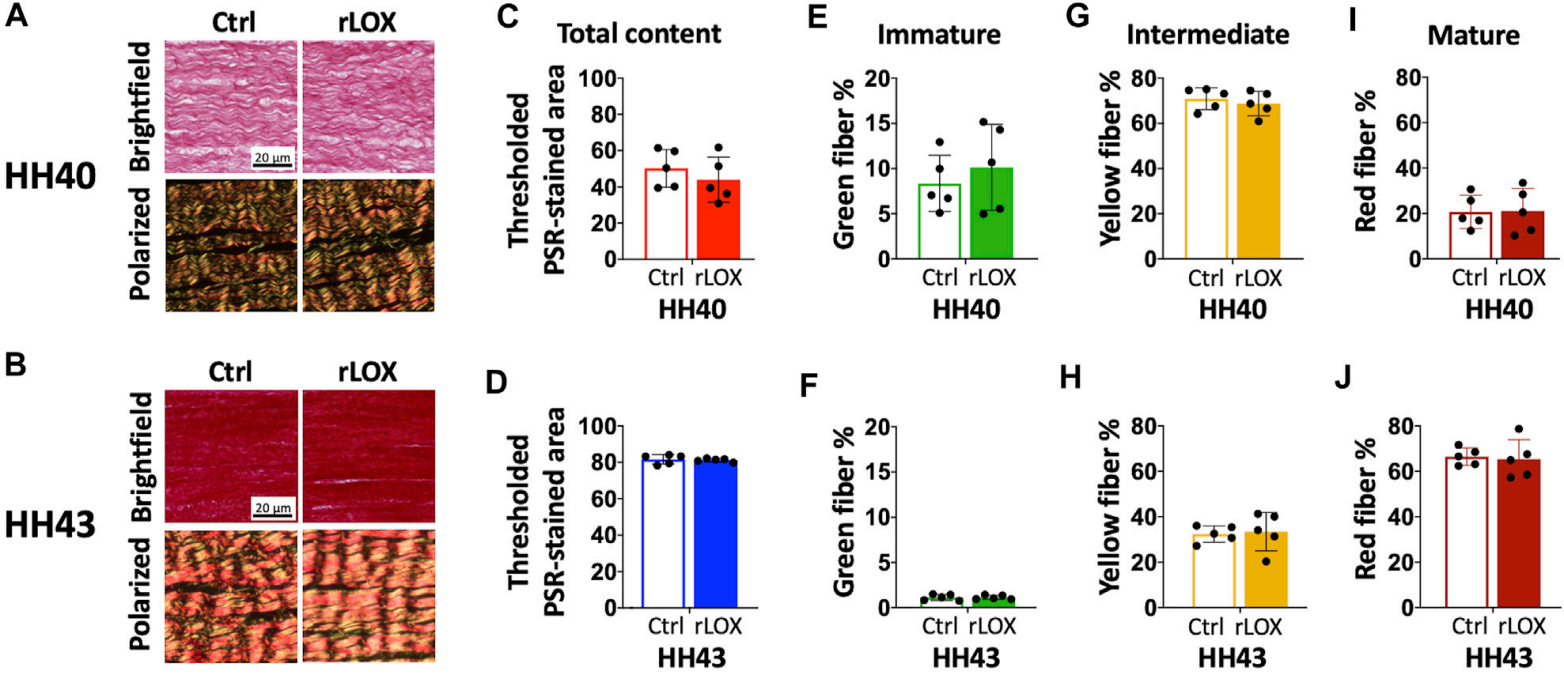
Collagen maturity of HH40 and HH43 tendon explants was not affected by rLOX treatment. **(A,B)** Representative brightfield and polarized microscopy images of PSR-stained HH40 **(A)** and HH43 **(B)** tendons that were treated with rLOX and Ctrl treatments. **(C,D)** Collagen content did not change with rLOX treatments in both HH40 **(C)** and HH43 **(D)** tendons. **(E–J)** Percentages of immature (green), intermediate (yellow), and mature (red) collagen did not change with rLOX treatments. **(E,F)** The percentage of immature collagen was not affected by rLOX treatments in both HH40 **(E)** and HH43 **(F)** tendons. **(G,H)** The percentage of intermediate collagen was not affected by rLOX treatments in both HH40 **(G)** and HH43 **(H)** tendons. **(I,J)** The percentage of mature collagen was not affected by rLOX treatments in both HH40 **(I)** and HH43 **(J)** tendons. Statistically significant differences were determined by Student *t*-test with *p* < 0.05. *n* = 5 per stage and treatment group.

Brightfield and polarized microscopy images of PSR-stained HH40 and HH43 tendon sections appeared similar between rLOX and Ctrl treatments (Figures 5A,B). Collagen content of both HH40 and HH43 tendons were not affected by rLOX treatment (Figures 5C,D), which agreed with our analyses of SHG images (Figures 4C,D). The percentages of immature, intermediate, and mature fibers in both HH40 and HH43 tendons also were not affected by rLOX treatments (Figures 5E–J).

**FIGURE 6:**
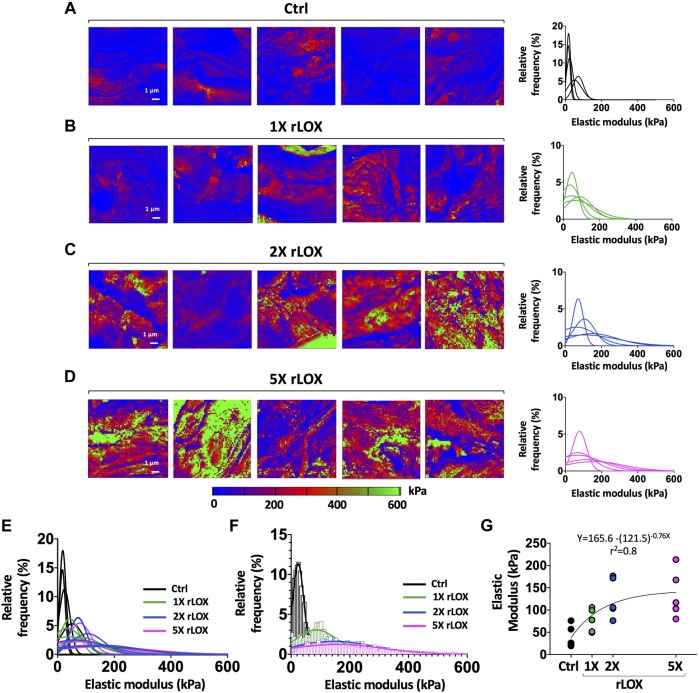
Plateau in elastic moduli of HH43 tendon explants was observed at higher rLOX concentration. **(A–D)** Elastic modulus maps and relative frequency histograms showed the shifts to higher elastic moduli between Ctrl (0X) **(A)** and 1X **(B)**, 2X **(C)**, and 5X **(D)** rLOX treatments. Treatment of 1X, 2X, and 5X rLOX led to wider distribution in the elastic modulus relative frequency histograms compared to Ctrl treatment. Relative frequency distribution of tendon elastic moduli almost overlapped between 2X and 5X rLOX treatments. The color scale of the elastic modulus maps was adjusted to capture the full range of maximum and minimum modulus values for all treatment groups. **(E)** All relative frequency distribution of tendon elastic moduli from 5 biological replicates from each treatment: Ctrl, 1X, 2X, and 5X rLOX. **(F)** Relative frequency distribution of tendon elastic moduli of one representative biological replicate from each treatment: Ctrl, 1X, 2X, and 5X rLOX. **(G)** Median tendon elastic moduli calculated from 4,096 data points per map increased significantly with rLOX treatments compared to Ctrl treatment. Tendon elastic modulus plateaued at higher rLOX concentration (exponential plateau fit, *r* ^2^ = 0.8). *n* = 5 per stage and treatment group. Note that the intensity bar for the heat map of elastic modulus in Panel has a different scale than in Panel 2 to capture the increases in elastic modulus with rLOX concentration.

AFM maps of HH43 tendons showed increasing regions of higher elastic modulus values with increasing rLOX concentrations (Figures 6A–D). Relative frequency histograms of the elastic moduli in these maps showed that elastic modulus distribution broadened and shifted to include higher elastic modulus values when treated with increasing rLOX concentrations (Figures 6A–F). Histograms of elastic moduli differed between tendons treated with 0X (Ctrl), 1X, and 2X rLOX (Figures 6A–C). In contrast, elastic modulus histograms appeared similar and overlapping for tendons treated with 2X and 5X rLOX, suggesting 2X and 5X rLOX had similar effects on elastic modulus (Figures 6C,D). Correspondingly, median elastic moduli of rLOX-treated tendons increased from 0X to 2X rLOX, but then appeared to plateau between 2X and 5X rLOX concentrations, reflected by a plateau curve fit with *r* ^2^ = 0.8 (Figure 6G).

**FIGURE 7:**
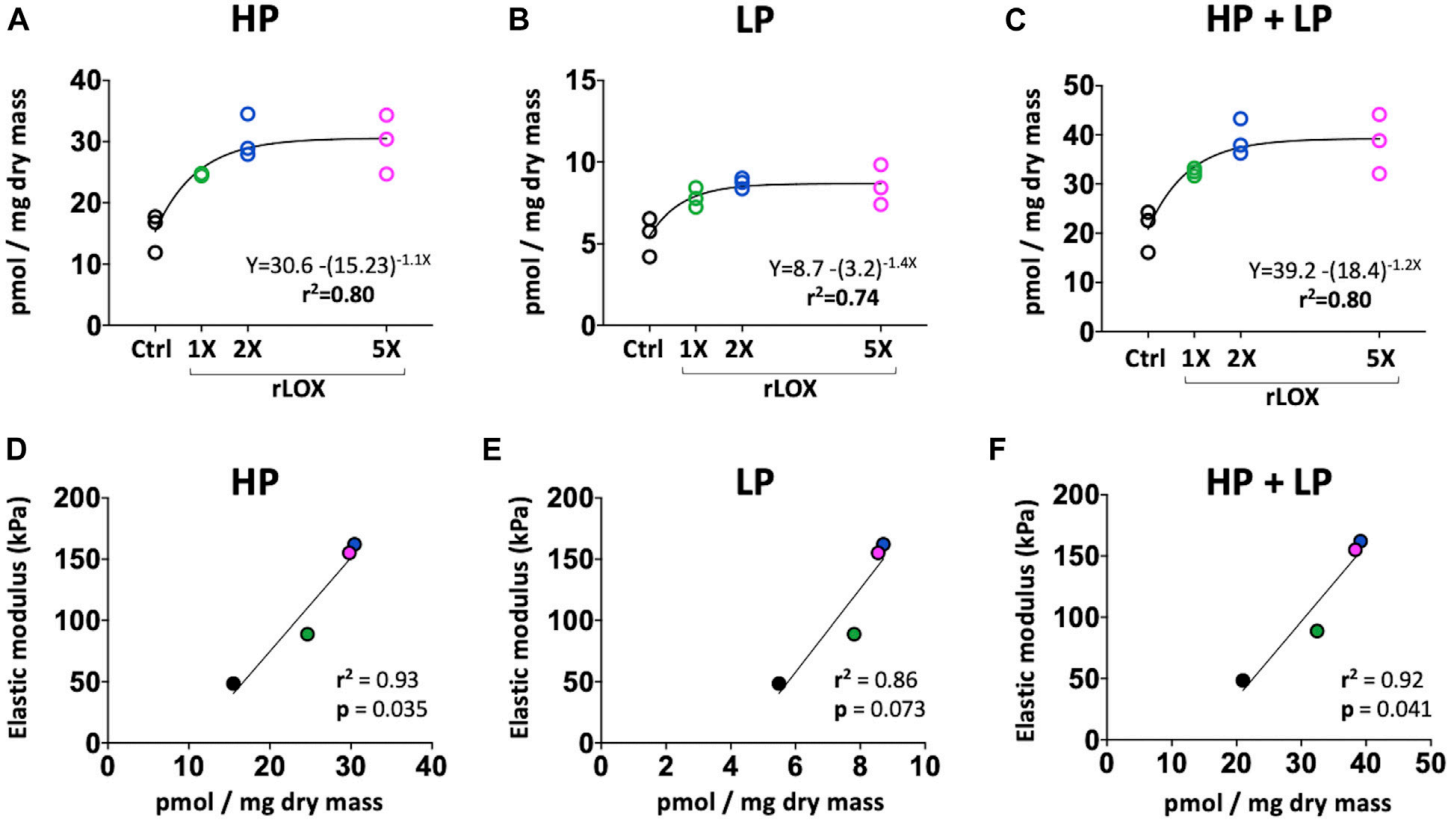
HP and LP crosslink density (normalized to dry mass). **(A–C)** HP **(A)**, LP **(B)**, and total HP and LP **(C)** crosslink density plateaued at higher rLOX concentrations (exponential plateau fit, *r* ^2^ = 0.7-0.8). **(D–F)** Correlation between LOX-mediated crosslink density and tendon elastic modulus. **(D)** HP crosslink density were highly and significantly correlated with tendon elastic modulus (*r* ^2^ = 0.93 and *p* = 0.035). **(E)** LP crosslink density showed high correlation with elastic modulus (*r* ^2^ = 0.86, *p* = 0.073). **(F)** Total HP and LP crosslink density were highly and significantly correlated with tendon elastic modulus (*r* ^2^ = 0.92 and *p* = 0.041). Statistically significant differences were determined by Pearson correlation and linear regression with *p* < 0.05. *N* = 3 per stage and treatment group.

HP, LP, and total HP and LP crosslink densities of HH43 tendons treated with 0X (Ctrl), 1X, 2X, and 5X rLOX were measured using HPLC. Similar to elastic moduli, crosslink densities increased from 0X to 2X rLOX, and then plateaued between 2X and 5X rLOX (Figures 7A–C). With and without rLOX treatment, HP crosslink densities were at least 4 to 5 times higher than LP densities, which was consistent with previous reports (Marturano et al., 2014). Notably, HP and total HP and LP crosslink densities correlated highly and significantly with tendon elastic modulus (*r* ^2^ = 0.93, *p* = 0.035; *r* ^2^ = 0.92, *p* = 0.041; respectively) (Figures 7D–F).

## Discussion

In this study, we demonstrated rLOX treatment can be used to controllably enhance the mechanical properties of developing tendons at different stages of functional tissue formation. Our exciting proof-of-concept data motivates the idea that a LOX-based therapeutic could be used throughout different phases of adult tendon healing to improve outcomes. As another application, a LOX-based intervention could potentially restore the mechanical properties of abnormally developing tendons during fetal development *in utero*. In this study, rLOX treatment increased tensile elastic modulus and peak strength in both HH40 and HH43 tendons. Nanoscale elastic modulus also increased significantly with rLOX treatment in both stage tendons. These changes in mechanical properties corresponded with changes in LOX-mediated crosslink density, whereas cell viability and collagen content and collagen fiber maturity did not change with rLOX treatment for either developmental stage. Interestingly, collagen fiber alignment in HH43 tendons was significantly enhanced by rLOX treatment. A particularly exciting finding was that crosslink density and tendon elastic modulus both plateaued at higher rLOX concentrations. The overlapping plateaus and the high correlation between elastic modulus and crosslink density (*r* ^2^ = 0.92; *p* = 0.041) may have been due to limited availability of neighboring lysine residues within the tendon. It is exciting to consider the possibility that this mechanism could prevent the tendon from over-stiffening by preventing over-crosslinking of collagen, though this requires further investigation. Our studies demonstrate the potential for LOX-based therapeutic approaches to enhance tendon mechanical properties *via* LOX-mediated crosslinking which occurs naturally in the body. These novel findings have significant implications for strategies to treat adult tendon injuries, connective tissue disorders, and tendon-related birth abnormalities.

Tensile mechanical properties of tendon were significantly enhanced with rLOX treatment *in vivo*. Previously, we demonstrated LOX-mediated crosslinking of collagen is a key regulator of developing tendon mechanical properties. In particular, inhibition of LOX activity during tendon development inhibited the formation of LOX-mediated crosslinks (HP, LP) during development, which in turn prevented increases in elastic modulus (Marturano et al., 2013; Marturano et al., 2014). Our current study now demonstrates rLOX treatment can induce additional collagen crosslinking and lead to increases in tendon elastic modulus and peak stress without affecting peak strain (Figure 1). This enables the tendon to sustain greater mechanical load without requiring a change in length. A LOX-based therapeutic would be attractive, as LOX-mediated crosslinking is a natural regulator of tendon mechanical properties in human and other species (Eyre et al., 1984; Ng et al., 1996; Thorpe et al., 2010). In contrast, other studies have explored strategies to enhance tendon mechanical properties using crosslinkers that do not naturally occur within the human body, including genipin, 1-ethyl-3-(3-dimethylaminopropyl) carbodiimide, glutaraldehyde, and ultraviolet light (Caliari et al., 2011; Fessel and Snedeker, 2011; Fessel et al., 2014; Green et al., 2017). Unfortunately, these crosslinkers are associated with significant limitations, including cytotoxicity and significant reductions in metabolic activity at concentrations needed to enhance tendon mechanical properties (Caliari et al., 2011; Fessel and Snedeker, 2011; Fessel et al., 2014; Green et al., 2017). As an alternative to these efforts, our data suggest a LOX-based therapeutic would have significant clinical implications as it could enhance tissue mechanical properties *via* a crosslinking mechanism that occurs naturally in human without affecting cell viability.

Tensile elastic moduli of tendons at different stages of development were significantly enhanced with rLOX treatment *in vivo* (Figure 1). These data suggest that rLOX treatment could be effective when delivered at different phases of new tendon formation. The ability to enhance tendon mechanical properties *via* rLOX treatment at both an earlier and later phase of new tissue formation could be beneficial for adult tendon healing strategies as well as interventions for abnormal development. In particular, rLOX treatments could be administered at varying phases of new tissue formation after injury to adult tendon, presenting the possibility of accelerating tendon healing and return to function. It was exciting to observe that even with less organized collagen ECM, HH40 tendons increased in elastic modulus and peak stress with rLOX treatment, suggesting less organized ECM during adult tendon healing could also benefit from rLOX treatment. While it may be advantageous to administer interventions for tendon-related birth abnormalities as early as possible, this would be dependent on the timing of detection, which varies widely depending on the syndrome. *In utero*, congenital hand anomalies in which tendons are underdeveloped, are detected as early as the end of the first trimester. However, other tendon-related syndromes, such as clubfoot, are typically detected later in the second and third trimesters (Browne, 1937; Quintero et al., 1999; Lee et al., 2002; Krakow et al., 2009; Kowalczyk and Felus, 2016; Bogers et al., 2019; Bekiou and Gourounti, 2020). Thus, the ability to treat tendon abnormalities at varying stages of tissue development is important. However, it is also important to note that rLOX treatment would likely need to be tailored to the phase of tissue formation. In response to rLOX treatment at the same concentration, tensile elastic modulus of HH40 tendons increased 136% whereas that of HH43 tendons increased 35% compared to control treatments (Figure 1). During normal development, collagen content increases rapidly from HH38 through the latest embryonic stages (Marturano et al., 2013; Marturano et al., 2014). Considering HH40 tendons possess 2.8-fold lower crosslink-to-collagen ratio compared to HH43 tendons (Marturano et al., 2014), HH40 tendons appear to possess more available collagen sites for crosslinking than HH43 tendons. In this case, rLOX treatment would catalyze more collagen crosslinking in HH40 than HH43 tendons, leading to a higher fold increase in elastic modulus of HH40 than HH43 tendons compared to their respective controls. Future studies would be needed to optimize rLOX effects at different phases of tissue formation in adult and embryonic systems. It would be beneficial to develop a LOX-based therapeutic that can be effective at more than one stage of tendon tissue formation during adult tendon healing as well as during tendon development *in utero*.

Our previous work showed tensile modulus and nanoscale modulus both increase as a function of developmental stage (Marturano et al., 2013; Navarro et al., 2022). Here, we treated tendon explants with rLOX and used AFM to measure changes in the values and spatial distribution of nanoscale moduli. Similar to tensile moduli, median nanoscale elastic moduli of both HH40 and HH43 tendons increased significantly with rLOX treatment (Figures 2E,F). Firstly, these results suggest the enhancements in tendon mechanical properties measured with tensile testing and AFM were due to rLOX-induced crosslinking rather than unknown systemic effects induced by rLOX. Secondly, AFM maps revealed rLOX treatment shifted the spatial distribution of nanoscale moduli toward both higher values as well as greater heterogeneity within a tissue (Figures 2A–D, 6A–F). The AFM probe is sensitive to small-scale components in the tissue, including cells, fibrillar collagen, and other ECM components (Marturano et al., 2013). Previous AFM measurements of embryonic tendon also revealed that increases in nanoscale moduli are spatially heterogeneous, highlighting collagen-rich regions that could be crosslinked by LOX and collagen-poor regions that would be minimally affected by LOX (Marturano et al., 2013). Consistent with this, the spatial heterogeneity of moduli across the tendon appeared to decrease when LOX activity was inhibited to block the formation of new crosslinks during development (Marturano et al., 2013). Taken together, rLOX treatment in this current study likely induced formation of new crosslinks in the regions that contain fibrillar collagen, and had little effect on regions that lacked available sites for collagen crosslinking. Consequently, elastic modulus would have increased in collagen-rich regions and been less affected in collagen-poor regions of the tendon. This heterogeneity was likely the source of the variabilities observed in AFM measurements both within a sample as well as between samples of the same stage and condition (Figures 2, 6).

The rLOX-induced increases in tensile modulus and nanoscale modulus demonstrate the potential for a therapeutic that promotes LOX-mediated crosslinking to promote tendon mechanical properties during new tissue formation, perhaps during adult tendon healing or fetal tendon development. A concern, however, is that a LOX-based therapeutic could lead to too many collagen crosslinks and consequently over-stiffen the tendon. To test this possibility, we treated explant tendons with increasing concentrations of rLOX (up to 5 times higher (5X rLOX) than the initial rLOX concentration tested) and used HPLC to characterize changes in crosslink density and AFM to characterize changes in mechanical properties. It was exciting to discover that increases in both crosslink density and median nanoscale modulus plateaued at higher rLOX concentrations, and that crosslink density and elastic modulus correlated linearly (Figures 7D–F). These findings suggest the plateau in elastic modulus at higher rLOX concentrations was due to the plateau in LOX-mediated collagen crosslink density. LOX catalyzes the conversion of lysine and hydroxylysine residues to reactive aldehydes within the telopeptide regions of collagen (Eyre et al., 1984; Kagan and Li, 2003). Crosslinking occurs when these reactive aldehydes are near enough to other aldehydes or lysine residues to spontaneously form covalent bonds. Thus, not only do lysine and hydroxylysine residues need to be available and accessible, but they also need to be in close physical proximity to other residues on adjacent collagen molecules, fibrils, or fibers for LOX-mediated collagen crosslinking to occur. It is possible that higher rLOX concentrations did not lead to additional crosslinks because there were no more available and accessible crosslinking sites in physical proximity. In the absence of additional crosslink formation, modulus also would not increase at the higher rLOX concentrations, which is what we observed (Figures 6, 7). It is intriguing to consider that this plateau may have occurred before the tendon could become overly stiff. In particular, 2X and 5X rLOX treatment of HH43 tendons led to an elastic modulus that was 280% higher than with control treatment (Figure 6). For comparison, normally developing tendon increases its elastic modulus 300% from HH43 to HH45 (the chick embryo hatches after HH45) (Navarro et al., 2022). It is also possible that rLOX induced cell behaviors that regulated, partially or wholly, the increases and plateau in tendon mechanical properties. Future studies will need to examine how the mechanical properties at plateau after rLOX treatment compare with the mechanical properties of non-treated healthy tendons, as the ability for tendon to intrinsically prevent over-crosslinking by LOX would be an enormous advantage for a therapeutic that promotes LOX-mediated crosslinking.

There was a smaller difference between AFM-determined elastic moduli of HH40 and HH43 tendons (Figure 2) compared to our previous study (Marturano et al., 2013). Here, we used AFM to probe sections of tendon bundles after *in vitro* culture with rLOX or vehicle for 3 days, whereas our previous work used tensile testing and AFM to characterize the mechanical properties of whole tendon (Marturano et al., 2013). It is possible that when freed from the larger tendon structure and sheath, the tendon bundle exhibited a lower elastic modulus, as detected by AFM indentation testing, because it was in a less constrained state. Additionally, it is possible that after 3 days of culture, there was a small but significant enough amount of water penetration to reduce the elastic modulus of the tendon bundle. These factors may have contributed to the smaller difference in AFM-determined elastic moduli of HH40 and HH43 tendons in the current study.

Collagen content and maturity for both HH40 and HH43 tendons were not affected by rLOX treatment (Figures 4, 5). Interestingly, rLOX treatment increased collagen alignment in HH43 but not HH40 tendons. Fibrillar collagen density was relatively low in HH40 tendons, with visible space between fibers in SHG images (Figures 4A,B). In contrast, HH43 tendons possessed higher fibrillar collagen density, with tighter packing and greater alignment in SHG images (Figures 4A,B). With more space to move around, collagen in HH40 tendons may have been less organized when crosslinked by exogenous rLOX. In this case, disorganized collagen matrix would persist in HH40 tendons. In HH43 tendons, however, collagen was already in more densely packed arrangements when exogenous rLOX was added. With little room to move around, new collagen may have incorporated into the highly organized network and further promoted alignment as density increased. Crosslinking by exogenous rLOX would further stabilize this collagen organization. Additional studies would be needed to test this hypothesis for the stage-dependent effect of rLOX treatment on collagen organization.

This study establishes the potential for a LOX-based therapeutic to enhance mechanical properties of tendon. However, to develop treatments for tendon healing, future studies should examine the effects of rLOX treatment on crosslink density and mechanical properties of adult tendons, both healthy and after acute or chronic injury. Future studies should also explore delivery strategies to target whole tendon and specific regions within tendon. We previously discovered elastin is not detected in embryonic tendons (Marturano et al., 2013), but future studies with adult tendon should consider how LOX-induced crosslinking of elastin may also affect tendon mechanical properties. It would also be important for future studies to examine how higher concentrations and longer durations of rLOX treatment time may affect tensile mechanical properties, crosslinking rate, and cell behavior. Here, we hypothesized that rLOX directly mediated new collagen crosslink formation, reflected by the significant increases in HP and LP crosslink density in both HH40 and HH43 tendons (Figure 7). However, it is also possible that rLOX induced cells to synthesize more LOX, which could also lead to increases in collagen crosslinking. Thus, it could be interesting to examine the effects of rLOX treatment on mechanical properties of acellular tissues. Based on our findings, a LOX-based therapeutic could be applied at different stages during fetal development to enhance elastic modulus of abnormally weak tendons associated with musculoskeletal disorders. Similarly, treatment could be applied at various phases of new tissue formation during adult tendon healing. However, in both cases, a LOX-based treatment to enhance tendon mechanical properties at different phases of tissue formation would require optimization, and also need investigation of sustainability and longer term effects. Taken together, the exciting data presented here demonstrate the potential to develop a therapeutic strategy based on naturally occurring LOX-mediated collagen crosslinking to restore mechanical properties and function after postnatal tendon injury and abnormal fetal tendon development.

## Data Availability

The original contributions presented in the study are included in the article/Supplementary Material, further inquiries can be directed to the corresponding author.
